# A Review: Fundamental Aspects of Silicate Mesoporous Materials

**DOI:** 10.3390/ma5122874

**Published:** 2012-12-17

**Authors:** Zeid A. ALOthman

**Affiliations:** Chemistry Department, P.O. Box 2455, College of Science, King Saud University, Riyadh 11451, Saudi Arabia; E-Mail: zaothman@ksu.edu.sa; Tel.: +966-1-467-5999; Fax: +966-1-467-5992

**Keywords:** mesoporous materials, sol-gel, surfactants, catalyst

## Abstract

Silicate mesoporous materials have received widespread interest because of their potential applications as supports for catalysis, separation, selective adsorption, novel functional materials, and use as hosts to confine guest molecules, due to their extremely high surface areas combined with large and uniform pore sizes. Over time a constant demand has developed for larger pores with well-defined pore structures. Silicate materials, with well-defined pore sizes of about 2.0–10.0 nm, surpass the pore-size constraint (<2.0 nm) of microporous zeolites. They also possess extremely high surface areas (>700 m^2^ g^−1^) and narrow pore size distributions. Instead of using small organic molecules as templating compounds, as in the case of zeolites, long chain surfactant molecules were employed as the structure-directing agent during the synthesis of these highly ordered materials. The structure, composition, and pore size of these materials can be tailored during synthesis by variation of the reactant stoichiometry, the nature of the surfactant molecule, the auxiliary chemicals, the reaction conditions, or by post-synthesis functionalization techniques. This review focuses mainly on a concise overview of silicate mesoporous materials together with their applications. Perusal of the review will enable researchers to obtain succinct information about microporous and mesoporous materials.

## 1. Introduction

The synthesis, characterization, and application of novel porous materials have been strongly encouraged due to their wide range of applications in adsorption, separation, catalysis, and sensors. The design, synthesis, and modification of porous materials are in some aspects more challenging than the synthesis of dense materials. Therefore, new strategies and techniques are continuously being developed for the synthesis and structure-tailoring of mesoporous materials [1,2,3,4,5,6].

**Figure 1:**
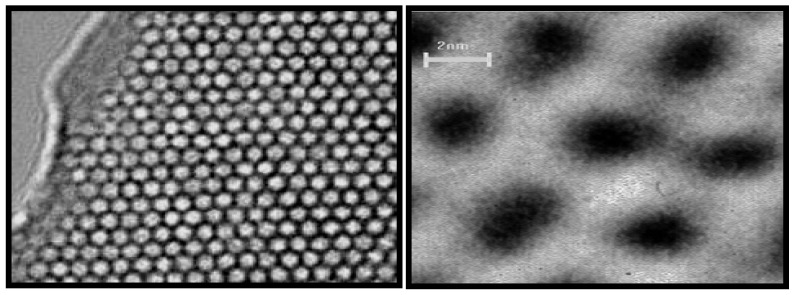
High resolution transmission electron microscopy (HRTEM) images of Mobile Crystalline Material (MCM-41) with hexagonal channels [3].

Ordered mesoporous materials, based on MCM-41 (Mobile Crystalline Material), are silicates obtained by hydrothermal synthesis and a liquid templating mechanism [1,2,3,4,5,6]. Such materials exhibit remarkable features such as pores with well-defined sizes and uniform shapes that are ordered to some degree over micrometer length scales to yield arrays of non-intersecting hexagonal channels. The latter structures are readily identifiable by transmission electron microscopy (TEM) images and X-ray powder diffraction (XRD) patterns (Figure 1). These materials possess high surface areas of about 1000 m^2^/g as revealed from surface area measurements. Mesoporous materials based on MCM-41 show excellent thermal, hydrothermal, and hydrolytic stabilities [7,8,9,10,11]. The walls of the channels are amorphous SiO_2_, and the porosity can be as high as 80% of their total volume [2,3,7]. These materials can be synthesized using anionic, cationic, or neutral surfactants or non-surfactant template pathways. The diameter of the channels (pores) can be controlled by changing the length of the template molecule. Moreover, changing the silica sources [e.g., fused silica, colloidal silica, tetraethylorthosilicate (TEOS)], surfactants [e.g., hexadecylamine (HDA), and cetyltrimethylammonium bromide (CTAB)], auxiliary compounds [e.g., 1,3,5-trimethylbenzene (TMB)], or reaction conditions (solvent, temperature, aging time, reactant mole ratio, and the pH of the medium) leads to the production of new mesoporous systems. At the same time, these changes also affect the thermal, hydrothermal, and mechanical stabilities of the materials [1,2,3,7].

Functionalization of the surface of these mesoporous materials with organic or inorganic functional groups leads to new physical and chemical properties [10]. These modified materials can be used in a variety of applications such as catalysis, adsorption, and separation as chromatographic column packing [12,13,14]. The materials have been characterized using several characterization techniques including X-ray powder diffraction (XRD), diffuse reflectance infrared Fourier transform spectroscopy (DRIFTS), scanning electron microscopy (SEM), transmission electron microscopy (TEM), elemental analysis (EA), thermogravimetric analysis (TGA), solid-state ^29^Si and ^13^C nuclear magnetic resonance spectroscopy (NMR), and surface area analysis including pore size, pore volume, and pore size distribution (PSD) measurements. In addition, the as-synthesized materials have been subjected to derivitization reactions in order to modify their surface with functional groups of interest. Their adsorption efficiency and selectivity have been determined along with their applications for separation of heavy and transition metal ions, radioactive materials, and organic compounds.

This review provides an introduction to the fundamental aspects of silicate mesoporous materials. It includes an overview and a concise historical introduction, a brief initiation to surfactant science, a broad introduction to sol-gel science, a general review of modification methods for MCM-41, and a summary of some applications of these materials. This review also includes introductions to the application of these modified materials for the adsorption and separation of toxic materials. The adsorption capacity, selectivity, and separation efficiency aree reported, and the effect of pH of the media, temperature, and time on the adsorption and separation is also covered. In addition, the competition effect of some metal ions of alkali and alkaline earth metals such as sodium (Na), potassium (K), magnesium (Mg), and calcium (Ca) with respect to the adsorption and separation of heavy metal ions and radioactive materials is reported. Various techniques were used in order to determine the adsorption and separation efficiency such as ultraviolet-visible spectroscopy (UV-Vis), inductively coupled plasma atomic emission spectroscopy (ICP), and atomic absorption spectroscopy (AAS).

## 2. Developments of Porous Materials

Zeolites and porous silicas take their place among the important porous materials for their wide applications in separation and catalysis. Zeolites are members of a large family of crystalline aluminosilicates. They were first discovered in 1756 by the Swedish scientist Cronstedt when an unidentified silicate mineral was subjected to heat; these strange minerals were found to bubble and froth, releasing bursts of steam. In the nineteenth century, zeolite minerals began to be well documented although there was a lack of general scientific interest. The term molecular sieve was derived from McBain in 1932 when he found that chabazite, a mineral, had a property of selective adsorption of molecules smaller than 5 Å in diameter [15]. In other words, molecular sieves retain the particles that fit within the channels and let the larger ones pass through. The term molecular sieves is used to describe a class of materials that exhibit selective sorption properties (*i.e*., that are able to separate a class of mixtures on the basis of molecular size and shape). However, Barrer and coworkers [16] studied the sorptive properties of chabazite and other porous minerals and reported that nitrogen and oxygen could be separated using a zeolite that had been treated to provide the necessary shape selectivity for discrimination between the molecular dimensions. Later, synthetic zeolites began to be used in large amounts for the production of pure oxygen from air. Between 1949 and 1954, Breck and coworkers [17] were able to synthesize a number of new zeolites (types A, X, and Y) which were produced in large scale to be used for the separation and purification of small molecules. Since then, the nomenclature of this kind of porous material has become universal. The success of synthesizing crystalline aluminosilicates, in particular the emergence of the new family of aluminophosphates [18] and silicoaluminophosphates [19], made the concept of zeolites and molecular sieves more complicated.

The small pore entrances (diameters) in zeolites (e.g., 0.4 nm in zeolite A) were attractive for commercial applications because they provided the opportunity for selective adsorption based on small differences in the size of gaseous molecules. In addition, these materials caught the attention of scientists who were interested in catalysis. At the beginning, the oil industry was reluctant to accept the idea, since it was thought that these materials had pores too small to be of interest for cracking activity (break down of long hydrocarbon molecules into gasoline and other useful products). The zeolite marketing prospects were improved when Breck and coworkers showed rare earth-containing zeolites had the ability to handle cracking activity [17]. There has been, however, a continually growing interest in expanding the pore sizes of zeotype materials from the micropore region to mesopore region in response to the increasing demands of both industrial and fundamental studies. Examples are the separation of heavy metal ions, the separation and selective adsorption of large organic molecules from waste water, the formation of a supramolecular assembly of molecular arrays, the encapsulation of metal complexes in the frameworks, and the introduction of nanometer particles into zeolites and molecular sieves for electronic and optical applications [20,21,22]. Therefore, to meet these demands, numerous experiments to create zeotype materials with pore diameters larger than those of the traditional zeolites were carried out. Since it was thought that most of the organic templates used to synthesize zeolites affect the gel chemistry by filling the voids in the growing porous solid, many of these attempts used larger templates. It was not until 1982 that success was achieved by changing the synthesis gel compositions when the first so-called ultra large pore molecular sieve, which contains 14-membered rings, was discovered [18]. Indeed, this not only broke the deadlock of the traditional viewpoint that zeolite molecular sieves could not be constructed with more than 12-membered rings, but also stimulated further investigations into other ultra large pore molecular sieves, such as VPI-5 with an 18-tetrahedral ring opening, cloverite, and JDF-20 [23,24,25]. While these zeolites attracted much attention and were of scientific importance, they have not found any significant applications because of their inherently poor stability, weak acidity, or small pore size (0.8–1.3 nm). As a consequence, they seem to be inferior compared to pillared layered clays.

Yanagisawa *et al.* described in the early 1990s the synthesis of mesoporous materials that have characteristics similar to that of MCM-41 [26]. Their preparation method is based on the intercalation of long-chain (typically C-16) alkyltrimethylammonium cations, into the layered silicate kanemite, followed by calcination to remove the organic species, which is later called surfactant, yielding a mesoporous material. The silicate layers condensed to form a three dimensional structure with nanoscale pores. ^29^Si solid-state NMR spectroscopy indicated that a large number of the incompletely condensed silica site Si(OSi)_3_(OH) (Q3) species were converted to the completely condensed silica site Si(OSi)_4_ (Q4) species during the intercalation and calcination processes. The X-ray powder diffraction gave only an uninformative peak centered at extremely low angles. Unfortunately, there were no further characterization data available which lead to disregard of the results of Yanagisawa *et al*.

In 1992, researchers at Mobil Corporation discovered the M41S family of silicate/aluminosilicate mesoporous molecular sieves with exceptionally large uniform pore structures [27] and later they were produced at Mobil Corporation Laboratories [28]. The discovery resulted in a worldwide resurgence in this area [1,2,3,7]. The synthesis of this family of mesoporous materials is based on the combination of two major sciences, sol-gel science and surfactant (templating) science. The template agent used is no longer a single, solvated organic molecule or metal ion, but rather a self-assembled surfactant molecular array as suggested initially [7,8,9,11]. Three different mesophases in this family have been identified, *i.e.*, lamellar (MCM-50), hexagonal (MCM-41), and cubic (MCM-48) phases [29]. The hexagonal mesophase, denoted as MCM-41, possesses highly regular arrays of uniform-sized channels whose diameters are in the range of 15–100 Å depending on the templates used, the addition of auxiliary organic compounds, and the reaction parameters [7,8,9,10,11]. The pores of this novel material are nearly as regular as zeolites, however, they are considerably larger than those present in crystalline materials such as zeolites, thus offering new opportunities for applications in catalysis, chemical separation, adsorption media, and advanced composite materials [11,28,29]. MCM-41 has been investigated extensively because the other members in this family are either thermally unstable or difficult to obtain [30].

In 1998, prominent research produced another type of hexagonal array of pores namely Santa Barbara Amorphous no 15 (SBA-15). SBA-15 showed larger pore size from 4.6 to 30 nm and discovery of this type of material was a research gambit in the field of mesoporous material development [31]. This SBA-15 mesoporous material has not only shown larger pores, but also thermal, mechanical and chemical resistance properties and that makes it a preferable choice for use as a catalyst. The formation of ordered hexagonal SBA-15 with uniform pores up to 30 nm was synthesized using amphiphilic triblock copolymers in strong acidic media was reported in the literature [32,33,34]. A detailed review on types, synthesis, and applications towards Biorefinery Production of this SBA 15 mesoporous material has already been published in the literature [35].

### 2.1. Definition and Classification of Porous Materials

**Figure 2:**
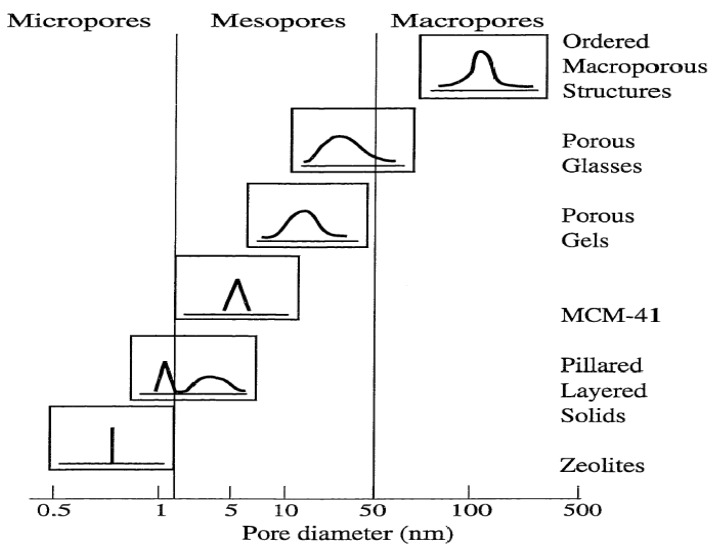
Schematic illustrating pore size distribution of some porous materials [39].

Porous materials created by nature or by synthetic design have found great utility in all aspects of human activities. Their pore structure is usually formed in the stages of crystallization or by subsequent treatment and consists of isolated or interconnected pores that may have similar or different shapes and sizes. Porous materials with small pore diameters (0.3 nm to 10 μm) are being studied for their molecular sieving properties. The pore shape can be roughly approximated by any of the following three basic pore models, (a) cylindrical (b) ink-bottled and (c) slit-shaped pores [36,37,38]. Depending on the predominant pore sizes, the porous solid materials are classified by IUPAC: Microporous materials, (1) having pore diameters up to 2.0 nm; (2) having pore sizes intermediate between 2.0 and 50.0 nm; and (3) macroporous materials, having pore sizes exceeding 50.0 nm (Figure 2) [39].

**Figure 3:**
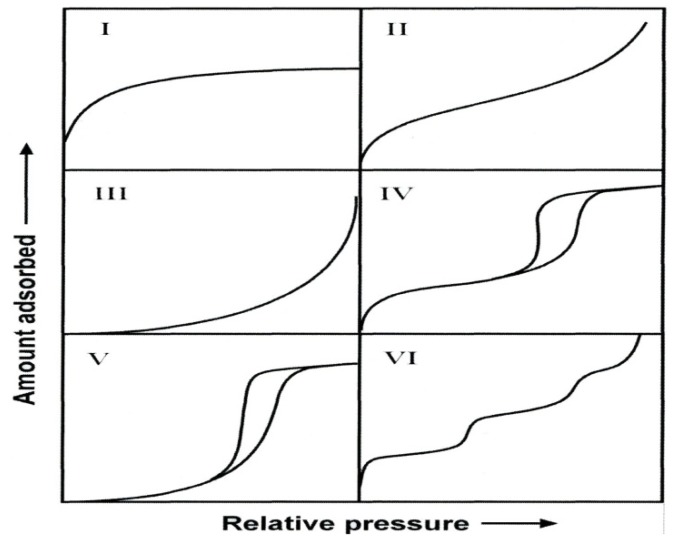
The IUPAC classification of adsorption isotherms showing both the adsorption and desorption pathways. Note the hysteresis in types IV and V.

As indicated, the pore size is generally specified as the pore width which is defined as the distance between the two opposite walls. Obviously, pore size has a precise meaning only when the geometrical shape is well defined. Porosity of a material is usually defined as the ratio of the volume of pores and voids to the volume occupied by the solid [36,37,38,39]. Porous materials are also defined in terms of their adsorption properties. The term adsorption originally denoted the condensation of gas on a free surface as opposed to its entry into the bulk, as in absorption. However, this distinction is frequently not observed, and the uptake of a gas by porous materials is often referred to as adsorption or simply sorption, regardless of the physical mechanism involved. Adsorption of a gas by a porous material is described quantitatively by an adsorption isotherm, the amount of gas adsorbed by the material at a fixed temperature as a function of pressure. Porous materials are most frequently characterized in terms of pore sizes derived from gas sorption data, and IUPAC conventions have been proposed for classifying pore sizes and gas sorption isotherms that reflect the relationship between porosity and sorption [36,37,38]. The IUPAC classification of adsorption isotherms is illustrated in Figure 3. The six types of isotherm (IUPAC classification) are characteristic of adsorbents that are microporous (type I), nonporous or macroporous (types II, III, and VI), or mesoporous (types IV and V) [36,37,38].

**Figure 4:**
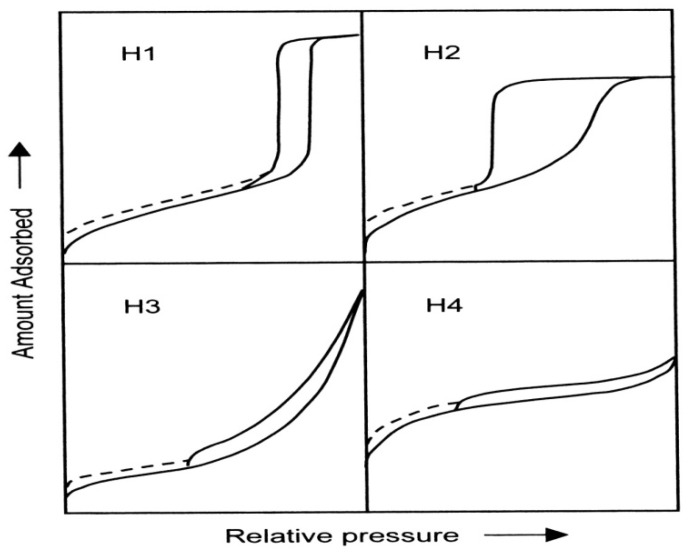
The relationship between the pore shape and the adsorption-desorption isotherm.

The adsorption hystereses in Figure 3 (IV and V) are classified and it is widely accepted that there is a correlation between the shape of the hysteresis loop and the texture (e.g., pore size distribution, pore geometry, and connectivity) of a mesoporous material. An empirical classification of hysteresis loops was given by IUPAC, which is based on an earlier classification of hysteresis by de Boer [36,37]. Figure 4 shows the IUPAC classification and according to IUPAC, type H1 is often associated with porous materials consisting of well-defined cylindrical-like pore channels or agglomerates of approximately uniform spheres. Type H_2_ ascribes materials that are often disordered where the distribution of pore size and shape is not well defined and also indicative of bottleneck constrictions. Materials that give rise to H_3_ hysteresis have slit-shaped pores (the isotherms revealing type H_3_ do not show any limiting adsorption at high *P*/*P*o, which is observed with non-rigid aggregates of plate-like particles). The desorption curve of H_3_ hysteresis contains a slope associated with a force on the hysteresis loop, due to the so-called tensile strength effect (this phenomenon occurs perhaps for nitrogen at 77 K in the relative pressure range from 0.4 to 0.45). On the other hand, type H_4_ hysteresis is also often associated with narrow slit pores [38].

The dashed curves in the hysteresis loops shown in Figure 4 reflect low-pressure hysteresis, which may be associated with the change in volume of the adsorbent, for example, the swelling of non-rigid pores or the irreversible uptake of molecules in pores of about the same width as that of the adsorptive molecule [38]. Porous materials can be structurally amorphous, paracrystalline, or crystalline. Amorphous materials, such as silica gel or alumina gel, do not possess long range order, whereas paracrystalline solids, such as *γ*- or *η*-Al_2_O_3_, are quasiordered as evidenced by the broad peaks on their X-ray diffraction patterns. Both classes of materials exhibit a broad distribution of pores predominantly in the mesoporous range. This broad pore size distribution limits the shape selectivity and the effectiveness of the adsorbents, ion-exchangers, and catalysts prepared from amorphous and paracrystalline solids. The only class of porous materials possessing narrow pore size distributions or uniform pore sizes comprises crystalline zeolites and related molecular sieves [40,41].

## 3. An Overview of Ordered Mesoporous Materials

*Meso*, the Greek prefix, meaning―in between, has been adopted by IUPAC to define porous materials with pore sizes between 2.0 and 50.0 nm [42]. Mesopores are present in aerogels, and pillared layered clays which show disordered pore systems with broad pore-size distributions. A constant demand has been developed for larger pores with well-defined pore structures. The design and synthesis of organic, inorganic, and polymeric materials with controlled pore structure are important academic and industrial research projects. Many potential applications require specific pore size, so that the control of pore dimensions to within a portion of an angstrom can be the dividing line between success and failure. Zeolites and zeolite-like molecular sieves (zeotypes) often fulfill the requirements of ideal porous materials such as narrow pore size distribution and a readily tunable pore size in a wide range. However, despite the many important commercial applications of zeolites, where the occurrence of a well-defined micropore system is desired, there has been a persistent demand for crystalline mesoporous materials because of their potential applications as adsorbents, catalysts, separation media or hosts for bulky molecules for advanced materials applications. Until the late 1980’s, most mesoporous materials were amorphous and often had broad pore size distributions. In the early 1990s, Kresge *et al*. [1] reported the emergence of a new family of socalled mesoporous molecular sieves, and in recent years, research in this area has been extended to many metal oxide systems other than silica and also to the novel organic-inorganic hybrid mesoporous materials [6].

**Figure 5:**
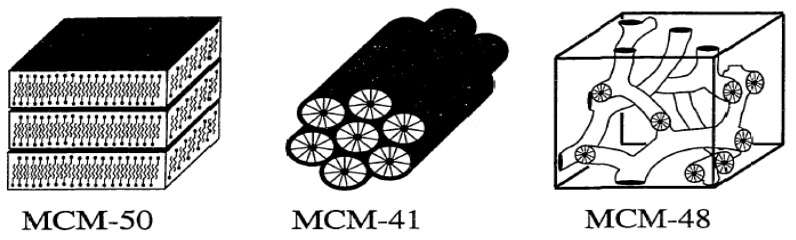
Schematic diagram of the M41S materials, MCM-50 (layered), MCM-41 (hexagonal) and MCM-48 (Cubic).

These new silicate materials possess extremely high surface areas and narrow pore size distributions [14]. Rather than an individual molecular directing agent participating in the ordering of the reagents forming the porous materials, assemblies of molecules, dictated by solution energetics, are responsible for the formation of these pore systems. This supramolecular directing concept has led to a family of materials whose structure, composition, and pore size can be tailored during synthesis by variation of the reactant stoichiometry, the nature of the surfactant molecule, the auxiliary chemicals, the reaction conditions, or by post-synthesis functionalization techniques. Figure 5 shows the different structures of the M41S family [42].

Following the initial announcement of MCM-41, there was a surge in research activity in this area [43,44]. Interestingly, di Renzo *et al*. [45] recently found a patent from 1971 in which a synthesis procedure similar to the one used by the Mobil group was described as yielding lowbulk density silica. The patent procedure was reproduced, and the product had all the features of a well-developed MCM 41 structure, as shown by transmission electron microscopy, X-ray diffraction, and nitrogen adsorption. However, in the original patent, only a few of the remarkable properties of the materials were actually described. It was the Mobil scientists who really recognized the spectacular features of these ordered mesoporous oxides.

Scientists have postulated that the formation of these molecular sieve materials is based on the concept of a structural directing agent or template. Templating has been defined as a process in which an organic species functions as a central structure about which oxide moieties organize into a crystalline lattice [20,46,47]. In other words, the template is a structure, usually organic, around which a material, often inorganic, nucleates and grows in a skin tight manner, so that upon the removal of the templating structure, its geometric and electronic characteristics are replicated by the inorganic materials [48]. The above definition has also been elaborated to include the role of the organic molecules such as: (a) space-filling species; (b) structural directing agents; and (c) templates [20].

In the simplest case of space filling, the organic species merely serves to occupy voids about which the oxide crystallizes. Therefore, the same organic molecule can be used to synthesize a variety of structures. Structural direction requires that a specific framework is formed from a unique organic compound, but this does not imply that the resulting oxide structure mimics identically the form of the organic molecule. In true templating, however, in addition to the structural directing component, there is an intimate relationship between the oxide lattice and the organic form such that the synthesized lattice contains the organic species fixed into position. Thus, the lattice reflects the geometry of the organic molecule.

**Figure 6:**
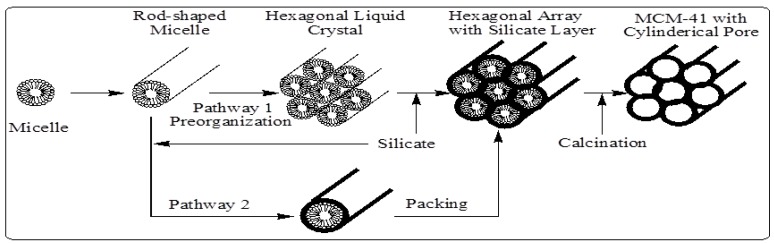
Schematic model of liquid crystal templating mechanism via two possible pathways [7].

In M41S materials, a liquid crystal templating (LCT) mechanism was proposed by the Mobil scientists in which supramolecular assemblies of surfactant micelles (e.g., alkyltrimethylammonium surfactants) act as structure directors for the formation of the mesophase (Figure 6). This mechanism behind the composite mesophase formation is best understood for the synthesis under high pH conditions. Under these conditions, anionic silicate species, and cationic or neutral surfactant molecules, cooperatively organize to form hexagonal, lamellar, or cubic structures. In other words, there is an intimate relationship between the symmetry of the mesophases and the final products [7,8,9,10,11]. The composite hexagonal mesophase is suggested to be formed by condensation of silicate species (formation of a sol-gel) around a preformed hexagonal surfactant array or by adsorption of silicate species onto the external surfaces of randomly ordered rod-like micelles through coulombic or other types of interactions. Next these randomly ordered composite species spontaneously pack into a highly ordered mesoporous phase with an energetically favorable hexagonal arrangement, accompanied by silicate condensation. This process initiates the hexagonal ordering in both the surfactant template molecules and the final product [7,8,9,10,11] as shown in Figure 6.

**Figure 7:**
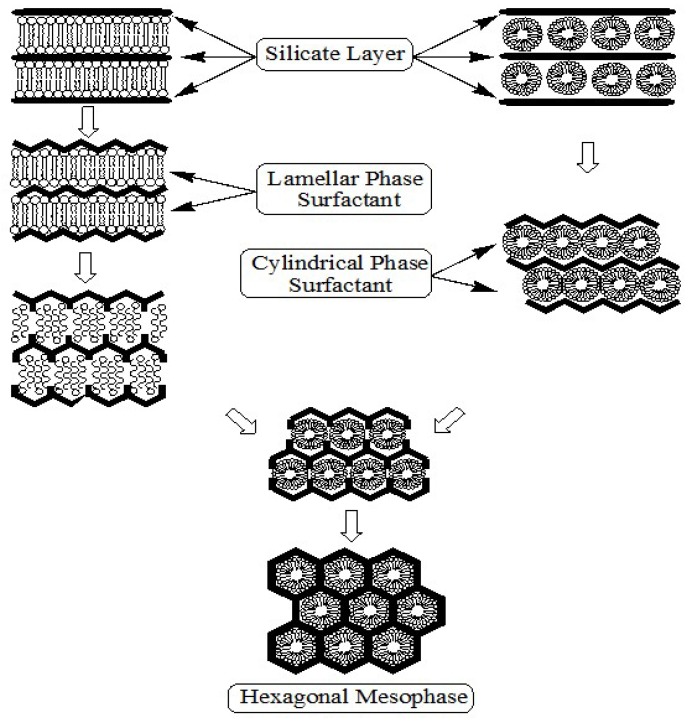
Schematic diagrams of the formation mechanism of MCM-41; (**a**) the proposed transformation mechanism by Monnier *et al*. [2] and (**b**) the formation mechanism proposed by Steel *et al.* [49].

Several other researchers further revised this liquid crystal templating mechanism. Chen *et al*. [49] studied the mechanism by carrying out *in situ* ^14^N NMR spectroscopy. They concluded that the randomly ordered rod-like organic micelles interact with silica species to form two or three monolayers of silica on the outer surfaces of the micelles. Then these composite species spontaneously self-organize into a long range ordered structure to form the final hexagonal packing mesoporous MCM-41. Moreover, they indicated that in the case of tetraethylorthosilicate as silica source, the concentration of the surfactant should be equal to or higher than the critical micelle concentration in order to obtain hexagonal MCM-41 materials. In addition to the previously proposed mechanism, there are two other suggested liquid-crystal template mechanisms. The first mechanism was put forward by Monnier *et al.* [2]. It was proposed that the surfactant is initially present in the lamellar phase regardless of the final product. This lamellar mesophase transforms to the hexagonal phase as the silicate network condenses and grows, see Figure 7a. The second mechanism was proposed by Steel *et al.* [50]. They suggested that, as the silicate source is introduced into the reaction gel, it dissolves into the aqueous regions around the surfactant molecules, and then promotes the organization of the hexagonal mesophase. The silicate first becomes ordered into layers between which the hexagonal mesophases of micelles are sandwiched. Further ordering of the silicate results in the layers wrinkling, closing together, and growing into hexagonal channels (see Figure 7b).

### 3.1. Chemistry of Surfactant/Silicate Solutions

**Figure 8:**
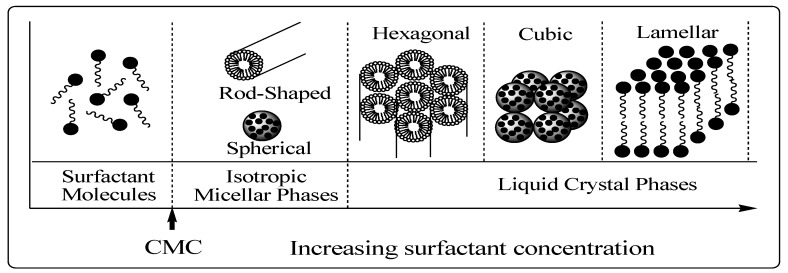
Phase sequence of surfactant-water binary system [37]. CMC = critical micellization concentration.

The structural phase of mesoporous materials (Figure 8) is based on the fact that surfactant molecules are themselves distinct as very active components with variable structures in accordance with increasing concentration [37]. At low concentrations, the surfactants energetically exist as monomolecules. With increasing concentration, surfactant molecules combine together to form micelles in order to decrease the system entropy [37,39,50]. This phenomenon is rationalized in the following way. Below the initial concentration threshold the monoatomic molecules aggregate to form isotropic micelles which is called the critical micellization concentration (CMC). In the micelle core, which is essentially liquid hydrocarbon, there is greater freedom for movement and so the entropy associated with the hydrocarbon tails also increases [39,51].

The ability of surfactants to reduce surface or interfacial tension is expected to be directly related to the CMC. As the concentration process continues, hexagonal close packed arrays appear, producing the hexagonal phases [51]. The next step in the process is the coalescence of the adjacent, mutually parallel cylinders to produce the lamellar phase. In some cases, the cubic phase also appears prior to the lamellar phase. The cubic phase is generally believed to consist of complex, interwoven networks of rod-shaped aggregates [52,53].

The formation of a particular phase in a surfactant aqueous solution at a given concentration depends not only on the concentrations but also on the nature of the surfactant itself, such as the length of the hydrophobic carbon chain, hydrophilic head group, and counter ion in the case of ionic surfactants. Moreover, it depends on environmental parameters, such as pH, temperature, ionic strength, solvent, and other additives (*i.e.*, organic compounds). Generally, the CMC decreases with the increase of the surfactant chain length due to the increase in the magnitude of the negative free energy change of micellisation. Increasing the ionic strength in the solution and increasing the valence of the counter ions lead also to a reduction in the CMC. On the other hand, the CMC increases with increasing counter ion radius, pH, and temperature. Also, it is known that non-ionic surfactants generally exhibit lower CMC’s than ionic surfactants [51,53].

It is important to note that a high surfactant concentration, high pH, low temperature, and low degree of silicate polymerization always support the formation of cylindrical micelles as well as the hexagonal mesophases [37,38].

**Figure 9:**
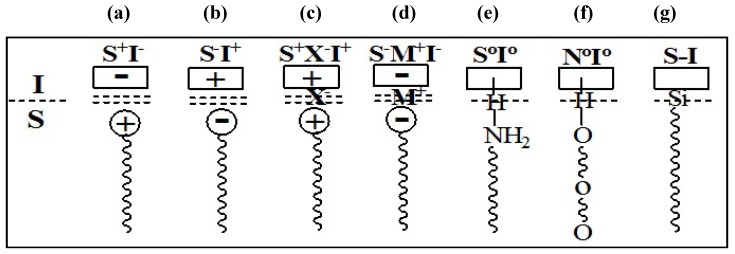
Interactions at the interface between the organic phase (S, N) and the inorganic phase (I) (**a**–**d**) ionic interactions; (**e**) and (**f**) hydrogen bonding; (**g**) covalent bond.

**Figure 10:**
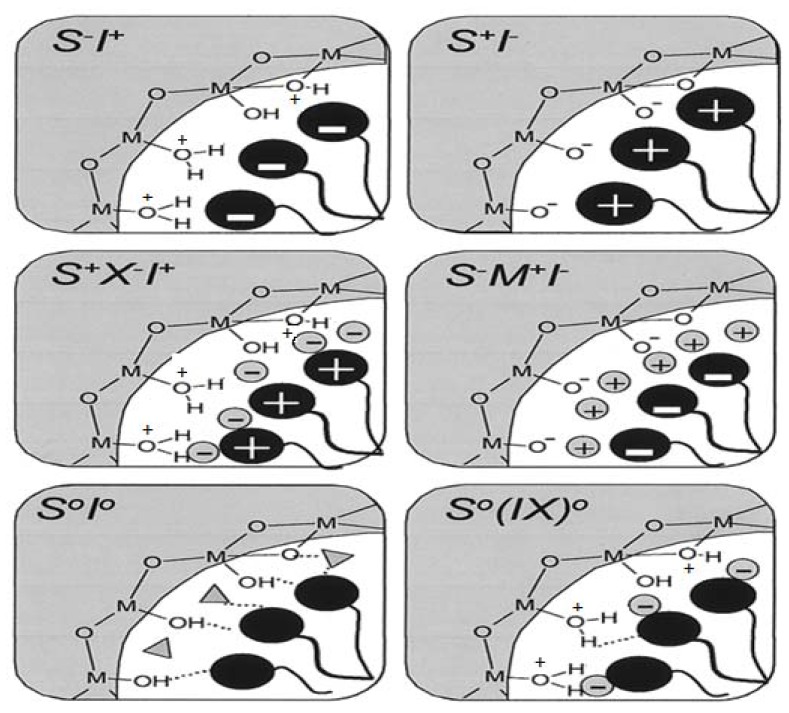
Schematic representation of the different types of silica-surfactant interfaces. S represents the surfactant molecule and I, the inorganic framework. M^+^ and X^−^ represent the corresponding counterions. Solvent molecules are not shown, except for the I°S° case (triangles); dashed lines correspond to H-bonding interactions [56].

The mesophases are formed by interaction of the organic parts with inorganic species, and thus both components play a crucial role in the assembly. The possible types of interactions between the organic and the inorganic parts that drive the formation of the mesophases depend on the charge on the surfactant, S^+^ or S^−^, on the inorganic species, I^+^ or I^−^, and on the presence of mediating ions, *i.e.*, X^−^ or M^+^. All permutations enabling Coulombic attraction are possible, *i.e.*, S^+^I^−^, S^−^I^+^, S^+^X^−^I^+^ or S^−^M^+^I^−^. Subsequently, three other pathways were also discovered. Neutral (S_o_) or nonionic (N_o_) species can interact with uncharged inorganic species by hydrogen-bonding (S_o_I_o_ or N_o_I_o_). Molecules with a covalent bond between the surfactant and inorganic parts were directly assembled (S-I), Figure 9 and Figure 10 illustrate the different interactions between the inorganic species and the surfactants. This formulation suggests the presence of a clearly defined interface between the organic and inorganic parts of the material [54,55].

The pore size in MCM-41 materials can be controlled by the hydrophobic alkyl chain length of the surfactants (altering the aggregation number and diameter) or with the aid of auxiliary organic compounds (*i.e.*, trimethylbenzene) as spacers and fillers. When the auxiliary organic species are added to the reaction gel, they are solubilized inside the hydrophobic regions of micelles, causing an increase in micelle diameter which leads to an increase in the pore size of the final product [57]. Strong electrostatic interactions between the ionic surfactants and the inorganic species result in an MCM-41 matrix with pore wall thickness that is influenced predominantly by the type of surfactant and only little by the pH conditions. Neutral template molecules, such as primary amines (with carbon tail lengths between C8 and C18), have also been employed to direct mesoporosity in silicates [54]. It was suggested that a neutral silicate would interact with micellar aggregates through hydrogen bonding between hydroxyl groups of hydrolyzed silicate species and the polar surfactant head-groups. The resultant framework structures were shown to have thicker silicate walls (*i.e.*, 1.5–3.0 nm) and therefore enhanced thermal and hydrothermal stability [55,56,58]. Other newly developed methods include the use of non-surfactant templates and copolymer precursor pathways [59,60,61]. The non-surfactant templated synthesis utilizes small organic molecules such as d-glucose, d-fructose, and dibenzoyl tartaric acid (DBTA) as the structure-directing agent [20]. By simply varying the concentration of the template molecules, mesoporous materials with different pore sizes can be obtained. The template can be easily removed by washing with water, solvent extraction, or calcination. These products possess high surface areas of ~1000 m^2^ g^−1^, pore volumes as large as ~1.0 cm^3^ g^−1^, and narrow pore size distributions. In addition to low cost, environmental friendliness, and easy removal of templates, this new approach also provides many other advantages such as mild synthesis conditions [62,63].

Since the discovery of these ordered mesoporous materials formed by the self-cooperative assembly of inorganic species and organic surfactants, researchers have aimed to understand and improve their structures to obtain forms suitable for application in adsorption, separation, catalysis, optical devices, and controlled polymerization inside the pores [64]. Mesoporous silica, in its many forms, adsorbs a wide range of compounds. For this reason it has been widely used in chromatographic columns for the adsorption and separation of chemical species.

## 4. An Overview of Sol-Gel Science Involved in the Synthesis of Mesoporous Silica

Organic/inorganic hybrid materials prepared by the sol-gel approach have rapidly become a fascinating new field of research in materials science. The explosion of activity in this area in the past two decades has resulted in tremendous progress in both the fundamental understanding of the sol-gel process and the development and applications of new organic/inorganic hybrid materials. Sol-gel chemistry has been investigated extensively since the 1970’s, when sol-gel reactions were shown to produce a variety of inorganic networks [65]. Sol-gel reactions are those which convert an aqueous metal alkoxide [Mn^+^(OR)*_n_*] solution into an inorganic network [65]. The sol-gel method is also capable of producing homogeneous, high purity inorganic oxide glasses at room temperature, much lower than the high temperatures required by the conventional glass manufacturing process. For example, silica can be obtained from melt processing glass, but the sol-gel method is more effective for the production of amorphous silica. Another advantage of the sol-gel procedure is its ability to produce silica in different forms such as molded gels [66], spun fibers [67], thin films [68], molecular cages [69], aerogels, xerogels [70], and mesoporous materials for a variety of applications such as gas, and liquid separations, optical coatings, protective films, membranes, and catalysis [71,72]. Therefore, changing the conditions of sol-gel polymerization and processing is helpful for controlling the bulk properties of silica. Among the advantages of using the sol-gel method is the availability of its raw materials in high purity. Modification of diverse properties of the inorganic network resulting from the sol-gel reaction is possible through the incorporation of the inorganic compound into different organic polymers.

**Scheme 1:**
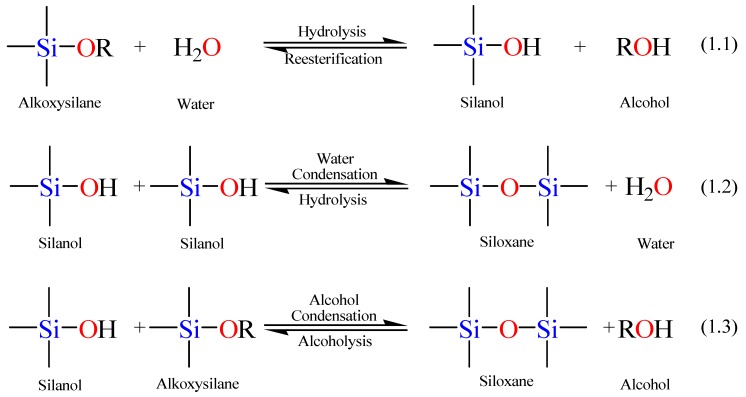
Sol-gel general reaction scheme.

The sol-gel process involves transformation of a sol to a gel [73]. A sol is defined as a colloid of small particles that are dispersed into a liquid. A gel, on the other hand, is a rigid non-fluid mass and is usually a substance made up of a continuous network including a continuous liquid phase [72,74,75,76]. Therefore, sol-gel reactions involve hydrolysis and condensation reactions of inorganic alkoxide monomers in order to develop colloidal particles (sol) and consequently convert them into a network (gel). A metal or metalloid element bound to various reactive ligands represents the precursor used to synthesize the colloids. Metal alkoxides are the reagents most used for this purpose due to their ease of hydrolysis in the presence of water. Alkoxysilanes, such as tetramethoxysilane (TMOS) and tetraethoxysilane (TEOS), are extensively used for the production of silica gels. Aluminates, titanates, and zirconates, however, are usually used for the synthesis of alumina, titania, and zirconia gels, respectively. Scheme 1 displays the involved hydrolysis and condensation reactions of TEOS. The hydrolysis step takes place by the addition of water to the TEOS solution under neutral, acidic, or basic conditions.

The hydrolysis step, Equation 1.1 in Scheme 1, leads to the generation of a silanol group (Si–OH). The mechanism of hydrolysis is catalyst dependent while its rate depends on the pH parameter, the water to alkoxide ratio, and the employed solvent. Since alkoxysilanes are not water-soluble, an organic co-solvent is required to facilitate the hydrolysis step by mixing the alkoxysilane with the water in the reaction mixture [76].

In the second step, the silanol group condense with either an alkoxide or another silanol group (the forward reactions in Equations 1.2 and 1.3 in Scheme 1) to build a strong siloxane linkage (Si–O–Si) with the loss of either an alcohol (ROH) or a water molecule. The siloxane hydrolysis and alcoholysis reactions (the reverse reactions in Equations 1.2 and 1.3, respectively) break the siloxane bond, but along with the forward reactions, the stepwise construction of the emerging network is permitted [72,74,76]. As the number of Si–O–Si bridges increases, the siloxane particles can aggregate into a sol, which disperses in the solution into small silicate clusters. Condensation of the latter silicate clusters leads to the formation of a network (a gel), trapping the water and the alcohol by-products. Removal of these trapped molecules from the formed gel network by heat treatment under vacuum yields a vitrified, dense glass network. It is noteworthy to mention that hydrolysis and condensation reactions go on concomitantly, so that the full hydrolysis of tetraalkoxysilane to Si(OH)_4_ does not necessarily occur before the beginning of the condensation reactions [72,77].

### 4.1. Water-to-Alkoxide Ratio

It has been found that the silica content of the formed gel increases upon increasing the water-to alkoxide ratio. Accordingly, one molecule of water is required for each alkoxide group to achieve full hydrolysis. Some researchers claimed that re-esterification would occur faster than the hydrolysis reaction in the case of using more than one molecule of water for every alkoxide group [76]. However, Schmidt and his coworkers worked over a wide range of water-to-alkoxide ratios and found no correlation between the water/alkoxide ratio and the achievement of complete hydrolysis [78]. The latter result is logically correct because water is generated *in situ* during the reaction.

The water-condensation step (Equation 1.2 in Scheme 1), on the basis of LeChâtelier’s principle, is anticipated to be hindered by increasing the water-to-alkoxide ratio. However, investigations of the impact of water-to-alkoxide ratio on the condensation step gave results contrary to the theoretical expectation. The condensation step was found to be accelerated upon increasing the water-to alkoxide ratio due to the increase in the solubility of silica and the increase in the concentration of the hydroxyl ion catalyst. Moreover, it was found that alcohol condensation to produce alcohol (Equation 1.3) was promoted upon employing a water:alkoxide ratio less or equal to 2, while water condensation was promoted at higher ratios [72,74,77]. The water-to-alkoxide ratio also influences the structure of the resultant gel network. It was established that high water/alkoxide ratios led to a more rigid gel network via prevention of contraction upon drying. The latter network rigidity was a result of the completion of hydrolysis and the occurrence of auxiliary condensation with the presence of a surplus amount of water [72,74,77].

### 4.2. Type and Amount of Catalyst

**Scheme 2:**
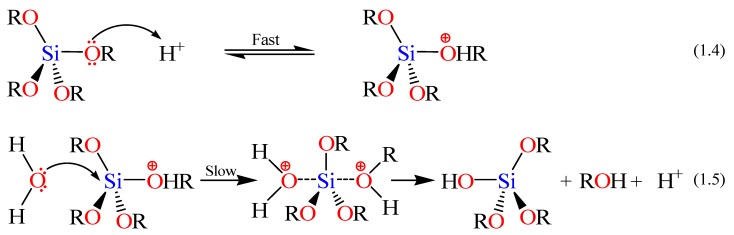
Hydrolysis mechanism of an alkoxysilane using acidic catalyst [77].

The rates and mechanisms of hydrolysis and condensation reactions are strongly affected by the identity of the catalyst. In acid catalysis (Scheme 2), the first step in hydrolysis (Equation 1.4) is electrophilic attack of the proton on an alkoxide oxygen atom, leading to the development of a positive charge on it. This electrophilic attack also makes the bond between the silicon center and the attacked oxygen (Si–O) more polarized and facilitates its breakage in the departure of the alcohol leaving group [79]. The rate-controlling step in acid hydrolysis (Equation 1.5) is an SN_2_ nucleophilic attack of water oxygen on the silicon from the backside. This latter nucleophilic attack results in the formation of a penta-coordinate transition state in which the silicon center is partially bonded to both –OH_2_ and –OHR. The incoming group (the attacking water molecule), the silicon center, and the leaving group (departing alcohol molecule) lie on an axis that is perpendicular to the plane of the silicon center and the other three alkoxide groups. It was also found that the hydrolysis reaction was first-order with respect to water concentration under acidic conditions. Accordingly, an increase in the water to alkoxide ratio resulted in an increase in the rate of hydrolysis. However, the enthalpy of the hydrolysis declined upon increasing extent of hydrolysis.

**Scheme 3:**
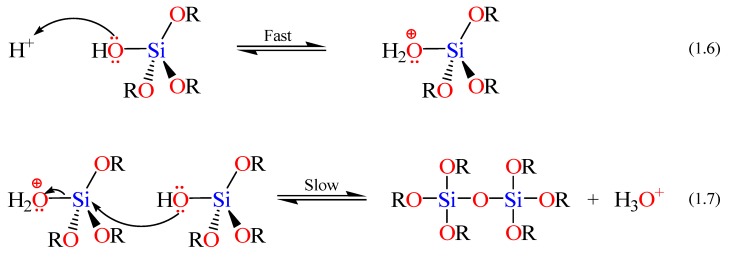
Condensation mechanism of an alkoxysilane using acidic catalyst [72,77].

The condensation rate and mechanism, as mentioned earlier, were found to depend on the pH of the reaction. For instance the condensation reactions (Equations 1.2 and 1.3 in Scheme I) become irreversible at low pH because the solubility of silica and its rate of dissolution are insignificant. The mechanism of condensation under acidic conditions is depicted in Scheme 3 (*vide infra*) [77]. The first step is the fast step and is an electrophilic attack of the proton on the oxygen of the silanol group. This attack results in the silanol oxygen becoming positively charged. The second step is the formation of a siloxane bridge via the loss of a hydronium cation (the catalyst) as a result of the condensation between a protonated silanol groups with an unprotonated one. Noticeably, the first steps in both hydrolysis and condensation reactions are similar.

**Scheme 4:**
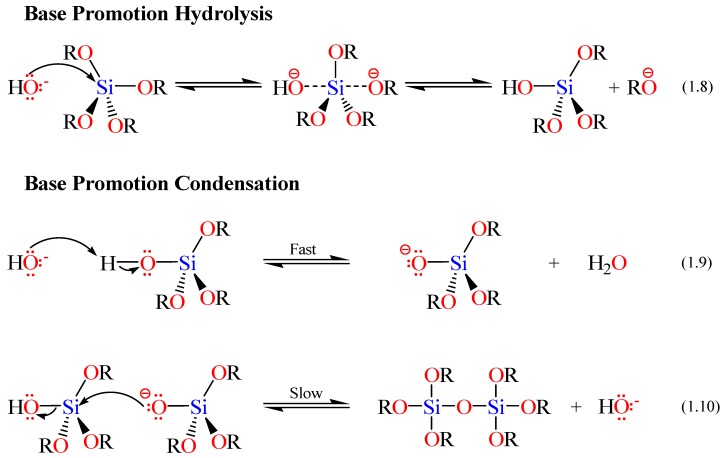
Hydrolysis and condensation mechanisms of an alkoxysilane using basic catalyst.

When a base catalyst is used for the formation of silica, the hydroxide ion serves as a nucleophile that attacks the silicon atom center of the tetraalkoxysilane in an SN_2_ hydrolysis step. The result of this step is a silanol and an alkoxide ion. Abstraction of the silanol proton by the hydroxide ion is the first step in the condensation process, leading to the formation of siloxide ion and water. A siloxane linkage is then formed through the SN_2_ attack of the latter ion on the silicon center of silanol. This step regenerates the hydroxide ion catalyst and is the rate-determining step of the condensation reactions. The hydrolysis and condensation reactions mechanisms are shown below in Scheme 4 [72].

**Scheme 5:**
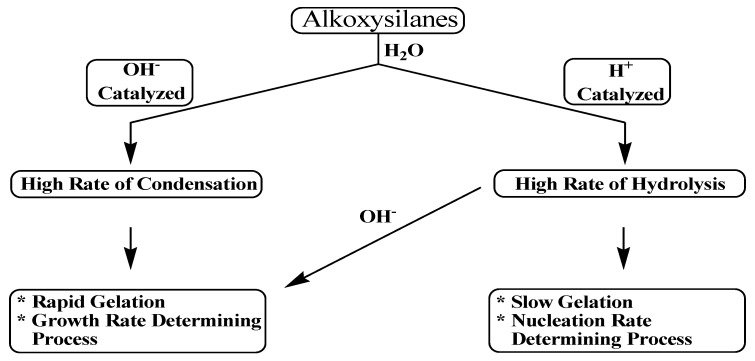
Effect of catalyst on hydrolysis and condensation.

Schmidt and coworkers [78] performed sol-gel reactions over a wide range of acid concentration. Their results showed no effect of the acid concentration on the structure of the resulting sol-gel. This conclusion was supported by ^29^Si NMR spectroscopy study which showed that the sol-gels obtained at different concentrations of the acid catalyst had similar spectra, indicating they had similar structures. However, McCormick and coworkers showed that a specific amount of the acid catalyst was necessary to initiate the reaction. Therefore, the existence of this minimum amount of catalyst allowed self-propagation. In addition, on the basis of gelation time and the fact that the condensation rate is inversely related to the gelation time, it was found that 0.07 M of acid resulted in the lowest condensation rate [72,74]. Most inorganic alkoxides hydrolyze and condense very rapidly in the absence of catalyst. In contrast, the hydrolysis of alkoxysilanes is so slow that it necessitates the addition of either an acid or base catalyst, see Scheme 5.

When an acid catalyst is employed, the rate-controlling step is the particle nucleation and the fast step is the hydrolysis. This fact leads to the production of more linear-like networks with less siloxane bonds and a high concentration of silanol groups, and hence, minimally branched polymeric species. On the other hand, alkoxide hydrolysis by base catalyst is faster than acid and prevents the quick aggregation of sol particles resulting in highly dense materials with fewer silanol groups in the overall network [72,74].

**Figure 11:**
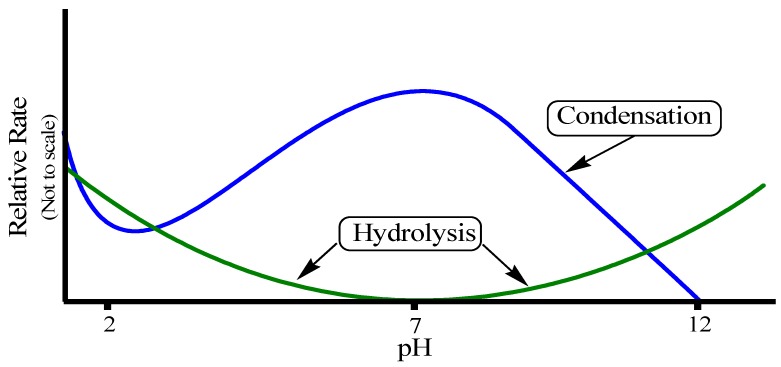
Effect of pH on hydrolysis and condensation rates.

The rates of both of the hydrolysis and condensation reactions depend strongly on the pH parameter as shown in Figure 11 [72,74,75,76]. For instance, at pH ≈ 7, molecular hydrolysis takes place at a slow rate, while molecular condensation occurs at a fast one. This inverse relationship between the rates of the hydrolysis and condensation reactions controls both the kinetics of the reaction and the ultimate network structure.

## 5. An Overview of Modification of As-Synthesized MCM-41

**Figure 12:**
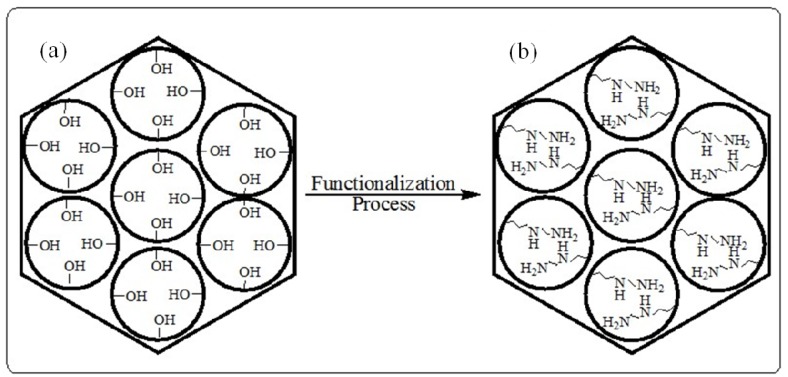
A diagram illustrating; (**a**) unmodified pore walls and (**b**) the presence of the functional groups on the pore walls.

Besides the extension from silicate to non-silica mesoporous materials, one other important way of modifying the physical and chemical properties of mesoporous silica materials has been by the incorporation of organic and inorganic components, either on the silicate surface, inside the silicate wall, or trapped within the channels. Figure 12 illustrates the functional groups in the internal pore surface.

Introduction of organic groups (functionalization) in the mesoporous materials permits the tuning of surface properties (e.g., hydrophilicity, hydrophobicity, acidity, basicity and binding to guest molecules), alteration of the surface reactivity, protection of the surface from chemical attack, hydrophobization of the surface by silylation to preclude water attack, and modification of the bulk properties of the materials while at the same time stabilizing the materials towards hydrolysis. Surface functionalized mesoporous materials are of great interest because of their potential applications in various areas such as catalysis, adsorption, chromatography, nanotechnology, metal ion extraction, and imprinting for molecular recognition [12,13,14]. For example, mesoporous silica having thiol groups on the pore surface showed high adsorption efficiency for heavy metals such as Hg, Ag, and Cd ions [80,81]. Sulfonic acid groups grafted onto mesoporous materials, as another example, exhibited high catalytic activity for selective formation of bulky organic molecules [82].

**Scheme 6:**
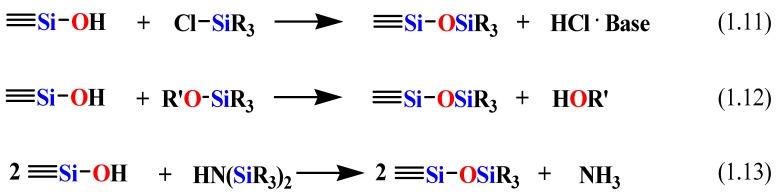
The silylation reaction for the modification of the surface of the mesoporous silica.

Mesoporous materials are interesting supports for organic functional groups due to their high surface area, large and uniform pore size, and narrow pore size distribution. While the silica framework provides thermal and mechanical stability, the surface organic moieties provide control of interfacial and bulk material properties such as flexibility and optical properties. Various literature reports describe methods for functionalizing the interior pore surfaces of mesoporous solids such as MCM-41 and SBA-15 [83,84,85,86,87,88,89,90,91,92,93,94,95,96]. These hybrid materials are generally synthesized via two methods [97,98]. The first one is the post-synthesis grafting method in which the pore wall surface of the pre-fabricated inorganic mesoporous materials is modified with organosilane compounds after the surfactant removal. The mesoporous materials possess silanol (Si–OH) groups that facilitate the attachment of the organic functions to the surface. Silylation is the most commonly used reaction for surface modification [96]. Moreover, esterification is another reaction used to carry out surface modification [7,27,99]. The silylation reaction method is achieved by one of the following reactions, shown in Scheme 6 [96].

The original structure of the mesoporous support is typically maintained after modification of the surface. Silylation occurs on all surface groups of the silica including the free or germinal silanols. However, hydrogen-bonded silanol groups are less accessible to modification because of the formation of hydrophilic networks [100]. In the post-synthesis grafting method, the host materials should be completely dried before adding modification precursors in order to avoid self-condensation of the precursors in the presence of water.

The second method for modification of the internal surface of the mesoporous materials is the direct synthesis. This method is based on the co-condensation of a tetraalkoxysilane (siloxane) and one or more organoalkoxysilane precursors with Si–C bonds through a sol-gel process. Siloxane precursors work as the main framework of the mesoporous materials while the organoalkoxysilane precursors contribute to the building of the framework and work as functional groups on the surface [84,85,87,88]. The direct synthesis has an advantage over the grafting method in which the former produces mesoporous materials with high loading of the functional groups [84,85].

Grafting of the mesopore surface with both passive [7,27,99,100] (*i.e.*, alkyl and phenyl) and reactive [83] (*i.e.*, amines, nitriles, thiol, halides, *etc.*) surface groups has been studied. The former can be used to tailor the accessible pore sizes and increase surface hydrophobicity while the latter to increase hydrophilicity and permit further functionalization. Multiple grafting has also been investigated. In order to minimize involvement of the external surface in reaction processes and to optimize selectivity, researchers have tried to graft to the external surface first through passive groups, before functionalizing the internal silanol groups [101]. Co-condensation using ionic [87], neutral surfactant [102], and non-surfactant templates [103] have all been demonstrated. Each of the two functionalization methods has certain advantages. If a uniform surface coverage with organic groups is desired in a single step, the direct method may be the first choice. It also provides better control over the number of organic groups incorporated in the structure. However, products obtained by post-synthesis grafting are often structurally better defined and hydrolytically more stable. Although pore size can be controlled to some extent by both methods, it is more easily achieved by grafting [84,85].

A recent development in functionalization of mesoporous materials has been the study of organic-inorganic species covalently bonded inside the mesoporous wall structure. The surfactant templated synthesis of these materials uses a precursor that has two trialkoxysilyl groups connected by an organic bridge [104,105]. The new technique allows stoichiometric incorporation of organic groups into silicate networks, resulting in higher loading of organic functional groups than by the grafting or direct synthesis methods. The only major problem with this approach is the lack of chemicals that have two trialkoxysilyl groups [104,105]. By introducing suitable functional groups onto the surface of these mesoporous materials, tenability of mechanical, surface chemical, electronic, optical, or magnetic properties of the hybrid composite may be possible [104,105].

## 6. Application of These Materials in Environmental Pollution Control Processes

Contamination of water streams by transition metals, heavy metals, and radioactive compounds (e.g., nickel, copper, lead, mercury, cadmium, uranium, and thorium) remains a concern in the field of environmental remediation. These materials enter the environment through a variety of avenues that include: mining, nuclear power plants, and industrial processing plants. Furthermore, some natural waters contain naturally high concentration levels of metals [106]. The presence of even low concentrations (ppb) of some heavy metals or radioactive substrates in natural water systems can have a harmful effect on both wildlife and humans. However, at these low concentrations of metal ions the sample often requires pre-concentration before analysis can be undertaken. Adsorption onto solid substrates (e.g., activated carbons, zeolites, aluminas, and silicas) provides one of the most effective means for adsorption, separation and removal of trace pollutants (heavy metal ions, radioactive compounds, *etc.*) from aqueous streams [10,12,13,106,107]. A wide variety of novel materials can be prepared by the chemical modification of ordered mesoporous materials, since numerous organic and inorganic functionalities can be used for this purpose [10,12,13,14]. In addition to their use in chromatographic separations, these materials have been increasingly used as heterogeneous catalysts in liquid phase organic reactions. It is their characteristics, such as viability and environmental safety, which makes them alternatives to traditional absorbent materials such as activated charcoal and zeolites. Their use as efficient materials for the selective adsorption and separation, and high capacity uptake of trace metals from aqueous systems is due to their unique characteristics such as high surface area, large pore size, and presence of reactive groups on the surfaces [106,108].

Many of the more recent advances have been focused on the use of modified silicas for clean technology. One area of research in which modified silicas are used for clean technology applications, other than catalysis, is in the adsorption, separation, removal, and analysis of trace components in aqueous systems. A wide variety of analytical techniques have been developed to separate and determine trace metal concentrations in natural water [12,13,14,106]. Several methods have been employed in the adsorption and separation of metal ions from aqueous solutions, such as activated charcoal, zeolites, clays, solvent extraction using a chelating agent [106] and the use of polymeric resins [107]. These methods suffer from a number of drawbacks. The use of activated charcoal, zeolites and clays showed low loading capacities and relatively small metal ion binding constants [108]. However, the use of chelating reagents (*i.e.*, iminodiacetate resin) is time consuming, whereas organic resins possess low surface area and low mechanical stabilities, and the time taken for the metal ion to be complexed, can be of the order of hours. Conventional methods such as precipitation are unfavorable especially when dealing with large volumes of matter which contain heavy metal ions in low concentration. Typically these ions are precipitated as hydrated metal oxides or hydroxides or sulfides using calcium oxide.

Precipitation is accompanied by flocculation or coagulation, and one major problem is the formation of large amounts of sediments containing heavy metal ions. In addition, these methods are often unselective towards the metal being analyzed, with interference from alkaline earth metals being particularly problematic [109]. In recent years, the use of modified mesoporous silica in the pre-concentration and separation of trace metal ions has been investigated [12,13,14,110]. Modified silica gels offer the advantages of high surface areas and increased chemical and mechanical stability. Nitrogen-containing organic groups have been shown selectively to bind to first row transition metals from solution [110]. Thus, Marshall and Mottola [109] prepared an immobilized quinolin-8-ol complex for the pre-concentration and separation of copper (II) ions. By varying the pH of the solution, a variety of transition metal (II) ions could be extracted selectively, even in the presence of alkali and alkaline earth metal ions. This makes the material useful for separation and analysis of trace metals in natural waters where alkaline earth metals are to be expected. There are factors that affect the adsorption and selectivity such as the pH and ionic strength of the water medium, the concentration ratio of the metal ion to the adsorbent, and the agitation time [111]. 

However, the unitary silica framework of siliceous MCM-41 limits its practical application, especially in catalysis owing to the lack of active sites. Therefore, great efforts have been focused on surface modification to expand the area of applications and many elements have been doped into the wall of MCM-41 including Al, Fe, Zn, Ti, V, Cu, Ni, W, and Mn [112,113,114,115]. Many researches have been focused on manganese oxides, owing to their ion-changing, molecular adsorption, catalytic, and magnetic properties and use as catalysts for environmental treatment of water. The detailed application of mesoporous materials as host-guest chemistry, environmental technology, adsorption, chemical sensors and electrode catalysis or adsorption is broadly reported in the published paper [116].

## 7. Conclusions

The literature reviewed revealed the fact that there has been a big increase in the production and utilization of microporous and mesoporous materials over the last few decades. The literature review also explains detailed systematic studies on these materials as well as some technical improvements in preparing and utilizing them. An overview of sol-gel science involved in the synthesis of mesoporous silica has been described. Functionalization of the surface of these mesoporous materials with organic or inorganic functional groups leads to new physical and chemical properties. These modified materials can be used in a variety of applications such as catalysis, adsorption, and separation as chromatographic column packing.

Introduction of organic groups in the mesoporous materials permits the tuning of surface properties, alteration of the surface reactivity, protection of the surface from chemical attack, hydrophobization of the surface by silylation to preclude water attack, and modification of the bulk properties of the materials while at the same time stabilizing the materials towards hydrolysis. Separation of transition metals, heavy metal ions or radioactive materials from aqueous streams is currently one of the most significant and fascinating problems to be challenged, severely hampered by the presence of a large excess of competing ionic species. Therefore, materials to be used for the adsorption and separation of these toxic substances are required to be specific enough to differentiate between transition metals, heavy metal ions and radioactive compounds on the one hand and on the other benign metal cations. A key issue for the applicability of these mesoporous materials is associated with the thermal, and more importantly the hydrothermal and mechanical stabilities.
